# Long-Term Stability Analysis of 3D and 2D/3D Hybrid Perovskite Solar Cells Using Electrochemical Impedance Spectroscopy

**DOI:** 10.3390/molecules25245794

**Published:** 2020-12-08

**Authors:** Sumayya M. Abdulrahim, Zubair Ahmad, Jolly Bhadra, Noora Jabor Al-Thani

**Affiliations:** 1 Center for Advanced Materials (CAM), Qatar University, Doha 2713, Qatar; sumayya@qu.edu.qa; 2 Qatar University Young Scientists Center (YSC), Qatar University, Doha 2713, Qatar; jollybhadra@qu.edu.qa (J.B.); n.al-thani@qu.edu.qa (N.J.A.-T.)

**Keywords:** perovskite solar cells, 3D & 2D/3D perovskite materials, electrochemical impedance spectroscopy, long-term stability, degradation

## Abstract

Despite the remarkable progress in perovskite solar cells (PSCs), their instability and rapid degradation over time still restrict their commercialization. A 2D capping layer has been proved to overcome the stability issues; however, an in-depth understanding of the complex degradation processes over a prolonged time at PSC interfaces is crucial for improving their stability. In the current work, we investigated the stability of a triple cation 3D ([(FA_0.83_MA_0.17_)Cs_0.05_]Pb(I_0.83_Br_0.17_)_3_) and 2D/3D PSC fabricated by a layer-by-layer deposition technique (PEAI-based 2D layer over triple cation 3D perovskite) using a state-of-art characterization technique: electrochemical impedance spectroscopy (EIS). A long-term stability test over 24 months was performed on the 3D and 2D/3D PSCs with an initial PCE of 18.87% and 20.21%, respectively, to suggest a more practical scenario. The current-voltage (J-V) and EIS results showed degradation in both the solar cell types; however, a slower degradation rate was observed in 2D/3D PSCs. Finally, the quantitative analysis of the key EIS parameters affected by the degradation in 3D and 2D/3D PSCs were discussed.

## 1. Introduction

Perovskite materials are one of the most trending contemporary photovoltaic research topics due to their rapidly evolving performance, which has already reached (at above 25%) very close to commercialized thin-film silicon solar cells. However, the lack of durability of these materials due to thermal instability and degradation upon exposure to humidity [1,2], UV radiation, and electric field [3] is still a major barrier to their commercialization. One of the most commonly studied 3D perovskites, MAPbI_3_, degrades under thermal stress due to the highly volatile MA+ ion [1]. Another thoroughly studied 3D perovskite, CsPbI_3_, demonstrates excellent thermal stability; however, it undergoes a phase change at room temperature to a form a large bandgap that is insufficient for absorbing UV radiation [4]. FAPbI_3_, on the other hand, also suffers from phase instability, as indicated by the presence of the yellow phase in the perovskite at room temperature [5]. Numerous studies have been performed to overcome the instability issues associated with perovskite materials. There have been attempts to modify three-dimensional (3D) perovskite by using additives [6], introducing intermediate phases [7], encapsulating the layers [8], etc. Several studies have been dedicated to analyzing the effect of mixed cations and halides to enhance PSCs’ stability and efficiency. Mixed cation perovskite is a unique way to benefit from the combined distinctive features of single cations: improved bandgap of perovskites with FA+ [9], impressive thermal stability with Cs+ [4], and high charge carrier mobility and long diffusion length with MA+ [5,10,11]. Incorporating MA cations with FA restricts the transition to the yellow non-perovskite phase by stabilizing the perovskite phase and enhancing the perovskite’s thermal and structural stability compared to the pure MA+ and FA+ cation 3D perovskite [12,13]. However, there is still the possibility of phase transition in the perovskite due to the large tolerance factor of FA [11] even in the presence of MA. Incorporating Cs has also proven to be a good choice due to its considerably smaller ionic radius and tolerance factor compared to FA and MA [11,14]. Hence, the addition of Cs to FA/MA cation results in the effective tuning of tolerance factor, and thus the resultant triple cation perovskite demonstrates enhanced performance and stability [11]. Saliba et al. [11] previously reported a mixed 3D perovskite precursor solution using triple cations—MA+, FA+, and Cs+—and managed to achieve a PCE exceeding 20% with moderate stability. Similarly, Singh et al. [15] also achieved an efficiency above 18% by using a triple cation perovskite powder for the PSC, which remained stable even after 2000 h.

Incorporating the 2D perovskite into 3D PSCs seems to be much more practical for enhancing stability while maintaining a high PCE [5,16,17,18,19,20,21]. A 2D perovskite has a general formula of R_2_(A)_n−1_B_n_X_3n+1_, where “R” represents a large-sized organic cation [5]. Introducing a bulky organic cation into the 3D perovskite layer’s crystal lattice passivates the vulnerable 3D perovskite against oxygen and moisture intrusion, resulting in enhanced stability of the PSC [3]. The compositional arrangement of 2D and 3D perovskite plays a significant role in the PSC’s resultant performance. Various techniques have been explored in the past to fabricate 2D/3D PSCs. There have been attempts at a single deposition technique to obtain a 2D/3D hybrid structure [20,21,22], as well as interfacial modification technique [23], a distinct layered 2D/3D technique [17], etc. The hybrid layered 2D/3D design demonstrated superior photovoltaic performance, reduced trap density, and prolonged PSC stability compared to the other methods. The choice of a 2D organic cation is crucial for enhancing the performance of the PSC. Phenylethyl ammonium (PEA+) and butylammonium (BA+) are the most commonly studied organic cations in 2D perovskites [5], and they have demonstrated an impressive enhancement in the performance and stability of PSCs. PEA+ is a bulky cation with a large radius that isolates the anionic layers of the 3D structure, transforming it into a 2D perovskite [17]. Cho et al. [18] investigated PEAI as the bulky cation in 2D perovskite and fabricated a layered 2D/3D structure and demonstrated an impressive PCE of 20.1% with 85% PCE retention after 800 h in ambient conditions. Similarly, Jiang et al. [24] also achieved a PCE of 23.32% owing to the superior passivation provided by the PEAI cation. Zhou et al. [25] also showcased a PCE of 21.4% with 1000 h impressive stability in ambient air conditions using PEAI.

**Figure 1:**
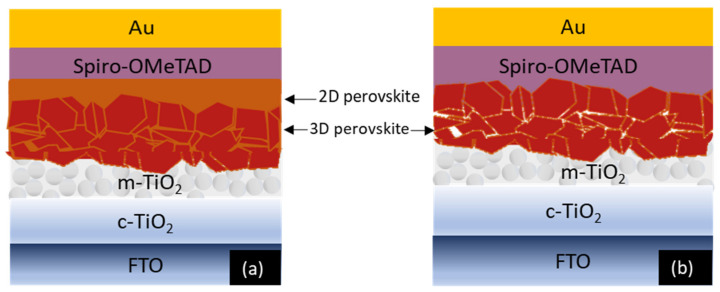
Schematic representation of 2D/3D perovskite solar cell (**a**) and 3D perovskite solar cell (**b**). The 2D perovskite layer is sandwiched between 3D perovskite and Spiro-OMeTAD (hole transporting material, HTM), preventing the HTM from diffusing across the 3D perovskite. Together with the HTM, the 2D perovskite acts as an additional protective layer against degradation due to the environmental parameters. The figures were drawn based on previously published literature [27] to explain the fact that the 3D perovskite commonly has irregularly packed grains, allowing for defects at the perovskite/HTM interface. Hence, the 2D layer improves the interface by deposing a uniform 2D layer over it.

Even though impressive stability results have been obtained with 2D/3D PSCs, despite the 2D layer passivation, the 2D layer can still degrade with aging, allowing the moisture to penetrate through to the 3D layer and damaging it. The 2D layer has been found to degrade under vacuum conditions due to the loss of 2D perovskite organic cation, which is mainly responsible for 3D layer passivation. Further comprehensive understanding of the interfacial processes at the 2D/3D heterojunction is yet to be developed [26]. Such an exciting direction of study can be critical in identifying fundamental degradation mechanisms, charge recombination, and built-in voltage losses and bringing improvements to PSCs’ performance and long-term stability. In this work, 3D and 2D/3D layered PSC structures with an initial PCE of 18.87% and 20.21%, respectively, were fabricated with a triple cation 3D perovskite and a PEAI bulky cation-based 2D perovskite. Results were gathered for 24 months, using current-voltage (J-V) and electrochemical impedance spectroscopy (EIS) measuring tools. The samples were kept under ambient room conditions. EIS proved to be a handy tool in analyzing the interfaces. Its unique capability to separate frequency-dependent resistive and capacitive responses district layers makes it very useful in the PSC field due to the multiple dynamic and complex processes occurring simultaneously at the interfaces. A time-independent EIS study is unreliable, as it does not prove whether the EIS spectra will stay consistent, and hence is not a true depiction of the system behavior. Therefore, the EIS tool was used to perform an in-depth degradation analysis by studying the system over a period, and a comparison of 3D and 2D/3D PSCs was established. 2D/3D and 3D perovskite solar cells (PSCs) were configured based on n-i-p structure, as shown in Figure 1a,b, respectively.

## 2. Results

**Figure 2:**
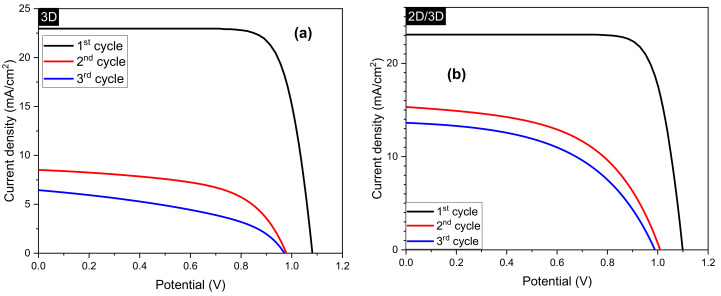

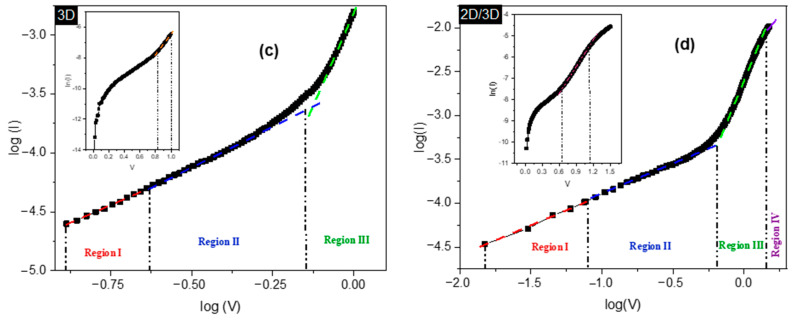
J-V characteristic curves of (**a**) 3D perovskite solar cell, and (**b**) 2D/3D perovskite solar cell. The aging measurements were divided into three cycles over 24 months of degradation with a time difference of 12 months. (**c**,**d**) Log-log plots of the 3D and 2D/3D samples under dark, respectively. The insets of (**c**,**d**) show the semi-log plots of 3D and 2D/3D samples.

Figure 2a,b show the J-V characteristic of 3D and 2D/3D PSC samples over a period of 24 months, divided into three cycles, with a time difference of 12 months. During the first 12 months, the 3D PSC decreased in J_sc_, PCE, and V_oc_ from 22.96 mA/cm^2^, 19.44% and 1.01 V to 8.51 mA/cm^2^, 4.75%, and 0.98 V, respectively (see Table 1). Subsequently, 3D PSC showed an overall reduction in J_sc_, PCE, and V_oc_ to 6.44 mA/cm^2^, 2.71%, and 0.97 V, respectively, within 24 months. The 2D/3D PSCs also showed a similar trend, with J_sc_ and PCE decreasing drastically from 23.08 mA/cm^2^ and 20.23% to 14.79 mA/cm^2^ and 7.85%, respectively, in the first 12 months. However, the rate of decrease in J_sc_ from 12 months onwards was negligible compared to 3D PSCs. The difference in V_oc_ of both the solar cell types was insignificant, and a similar observation was made in our previous work [28]. The 2D/3D PSC clearly shows a slower degradation rate which may be due to the protection from the 2D capping layer against environmental parameters. Moreover, the slope near the J_sc_ point, which is attributed to shunt resistance, was observed to be decreasing for both the solar cells. With aging, the junctions can become weak which can enhance recombination and hence decrease shunt resistances as observed in Figure 2a,b. Overall, 2D/3D-based PSCs were noted to have a higher PSC (6.83%) than that of 3D PSC (2.71%), even after 24 months. Similar is the case with the rest of the parameters, i.e., J_sc_, V_oc_, and FF. With the progression of time, this trend was seen to continue for all the parameters.

To analyze the conduction mechanism, log-log, and semi-log curves for J-V characteristics were plotted (see Figure 2c,d). Figure S1 exhibits log-log plots of 3D and 2D/3D samples after 3 months and 24 months. The log-log plots consist of three and four distinct regions in 3D and 2D/3D, respectively (in the voltage range 0 V–1.2 V, close to V_oc_), which help understand voltage-dependent conduction in both 3D and 2D/3D PSCs. It was noted that the current flows in both types of solar cells followed the power law, I∝V^m^, where “m” is the slope of the log-log plot. Mark and Helfrich reported that the current and the slope of J-V characteristics are affected by the magnitude of traps [29]; hence, each region of the log-log plot was calculated to analyze the trap characteristics of the PSCs as listed in Table 2. The log-log plots of both types of the cells, initially, followed a linear behavior (slope ~ 1) representing ohmic conduction. Region II and Region III slopes represent the presence of shallow and deep traps, respectively. These regions are typically associated with trapping and de-trapping of charge carriers, and the current in this region is assumed to be dominated by the trapped charge limited current (TCLC) model. Region IV (in 2D/3D) is associated with the space charge limiting current (SCLC) region in the potential range close to the V_oc_. It is assumed that in 2D/3D samples, the traps are now being filled, and the injected charge carriers are dominated by the SCLC. The slope of this region is found to be close to ~2, representing I ∝ V^2^. Next, to analyze the quality of the device, the ideality factor (n) was calculated. Ideality factor increases with an increase in traps [30]. The ideality factor emerges at the exponential regime of the J-V plot. Therefore, a semi-log curve was plotted, and the ideality factor was calculated using the slope of the linear region, and the values are listed in Table 1. The ideality factor of both 3D and 2D/3D PSCs was observed to be increasing with aging, indicating degradation in the structural quality. It should be noted that this technique only gives us information about the trap characteristics and the quality of cells; however, it is difficult to get in-depth insight regarding the degradation in the complex and multilayered PSC structure. Therefore, electrochemical impedance spectroscopy (EIS) was performed to get an in-depth analysis of the dynamic processes occurring within the bulk and interfaces of both 3D and 2D/3D PSCs.

**Figure 3:**
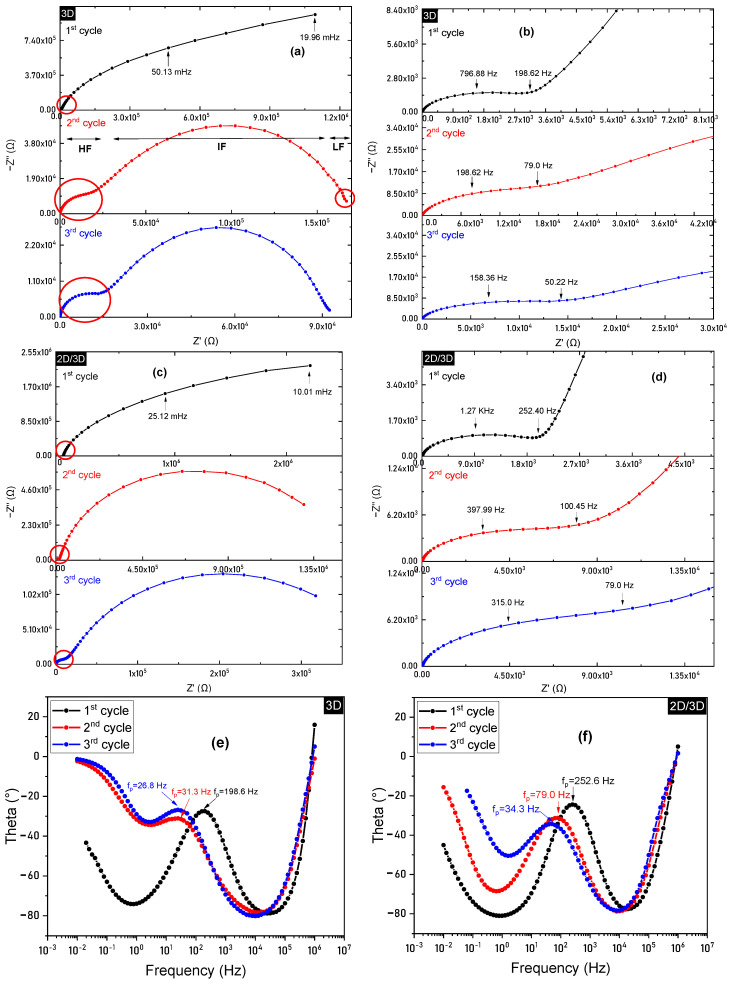

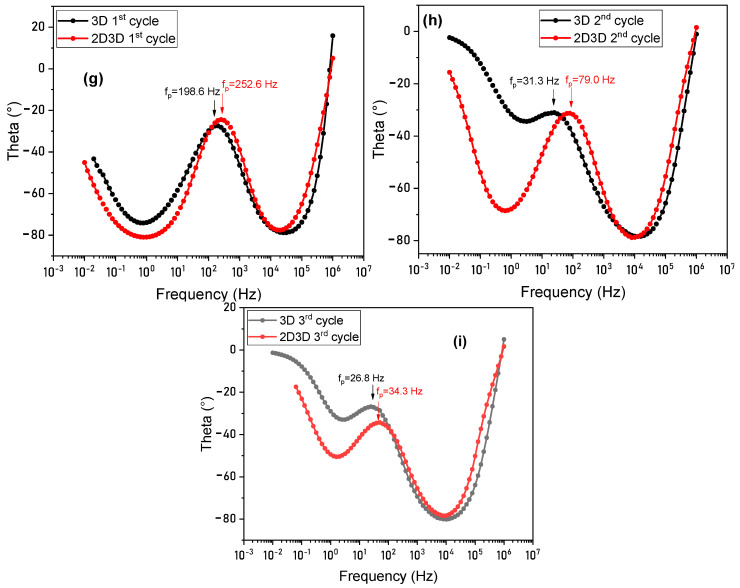
The evolution of electrochemical impedance spectroscopy (EIS) spectra of (**a**) 3D and (**c**) 2D/3D PSCs, measured after 3 months (1st cycle), 12 months (2nd cycle), and 24 months (3rd cycle). (**b**,**d**) EIS trend of 3D and 2D/3D PSC zoom-in of the red circled part. Some key frequency parameters are marked in the Nyquist plot. (**e**,**f**) Evolution of Bode plots over 24 months of 3D and 2D/3D samples. Bode plots of 3D and 2D/3D PSCs at (**g**) 3 months (1st cycle), (**h**) 12 months (2nd cycle), and (**i**) 24 months (3rd cycle). The peak frequencies (fp) are marked in Bode plots. The EIS measurements are not stable when taken on fresh cells due to perovskite instability during the EIS measurements. Therefore, the first EIS measurement was taken after 3 months of degradation of the samples instead of fresh cells, followed by 12 months and 24 months of measurement to analyze the aging process.

Electrochemical Impedance Spectroscopy (EIS), a non-destructive technique, was used to analyze the PSCs’ interfacial dynamic processes and electron/hole transfer kinetics. The experiment was carried out in the dark and short-circuit conditions at a small 10 mV AC perturbation. In the dark and at a low potential, this study ensured the stability of the devices during EIS measurements [31]. For data interpretation, electrical equivalent circuit models (EECs) were developed, representing resistive and capacitive elements linked to the electronic and ionic responses produced by PSCs within the specified frequency ranges [32]. Figure 3 shows the Nyquist plot progression with a time interval of 3 months (1st cycle), 12 months (2nd cycle), and 24 months (3rd cycle). The Nyquist plots consist of three distinct semicircles, a small arc at higher frequencies (HF) representing the range > 100 KHz, followed by a larger arc at intermediate frequencies (IF), representing the range 100 kHz–100 Hz. Additionally, in the lower frequency (LF) region (<1 Hz), a small arc can be visualized, which is not very clear in all of the Nyquist plots of Figure 3a (panel b), which may be due to the merging of time constants in that region. All the three regions are clearly marked in Figure 3a (panel b) for the better understanding of readers. The HF region is linked to interfacial capacitance and resistance at the Au/HTM/perovskite interface [33]. The IF region corresponds to resistive and capacitive elements relating to charge transport, recombination processes, while the LF region is related to the ionic diffusion process [33]. In the current study, notable changes in the impedance spectra were observed for both the solar cell types with aging. This paper focuses particularly on the evolution of the IF region behavior (recombination process) of 3D and 2D/3D PSCs over a period of 24 months.

**Figure 4:**
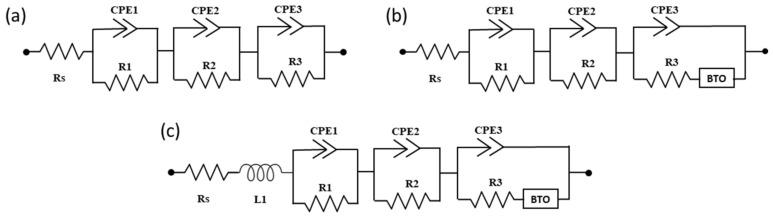
Electrical equivalent circuit (EEC) models analyzed for 3D and 2D/3D PSCs fitting. (**a**) 3RC model, (**b**) 3RC-BTO model, and (**c**) 3RC-BTO-Ls model. BTO is the Bisquert transmission line model used for low-frequency region fitting to consider the large dielectric effect. The EEC model proposed is attributed to dark and short circuit conditions. The models need to be modified depending on applied potential and light intensity.

To fit the obtained EIS spectra, a 3RC model was used, as shown in Figure 4. A detailed electrical equivalent circuit (EEC) analysis deduced that a simple R|C circuit could model the HF impedance response. In contrast, EEC fitting of the LF region was done by the Bisquert transmission line model to account for the giant dielectric effect at low frequencies [34]. Moreover, it was realized that implementing an inductor in the HF region enhanced the fitting of the impedance data. In our previous work [28], an extra RC had to be added for 2D/3D fitting. However, adding BTO to the EEC model enhanced the overall fitting in the current work and excluded the need to add an extra RC for 2D/3D fitting.

In the chosen model (Figure 4), CPE_1_ represents the geometric capacitance of the HF region. It is associated with the perovskite layer’s dielectric properties or the HF components of the contact layer i.e., Au/HTM/perovskite interface [35,36]. CPE_2_ is related to recombination in the IF region at the perovskite interface, while the CPE_3_ is associated with the ionic migration in the LF region [36]. Constant phase elements (CPEs) with an impedance Z_CPE_(jω) = 1/CPE_T_(jω)^CPE^ were used instead of ideal capacitors. This is because a perfect semicircle was not obtained in any of the spectra [37]; instead, depressed/flattened arcs were found during the EIS measurements. This effect is generally linked to various factors, including non-homogeneous roughness and porosities, presence of surface states, and non-ideal chemical capacitance of the device. In an ideal scenario, i.e., CPE_P_ = 1, this element is replaced with a pure capacitor. However, in the current scenario, the initial CPE_P_ values for the capacitive elements were close to ~0.9 (which is typical for a real device); therefore, they had to be replaced by CPEs. The CPE_P_ values decreased further with time, owing to the degradation of devices. R_s_ in the specified configuration are attributed to the series resistance associated with the FTO substrate. Owing to the inverse relationship between R_rec_ and recombination rate, a higher value indicates a noteworthy decrease in the recombination rate and subsequent improvement in electrons’ induction to the photoanode and J_sc_ [38]. On the other hand, the R_ct_ resistive element is related to Au/HTM/perovskite interface; hence, the HF arc (and therefore the R_ct_ resistance) is greatly dependent on the contact transport resistance [35].

Table 3 presents the values obtained from evaluating multiple EEC models used to fit the 3D and 2D/3D PSCs’ EIS spectra. At the initial characterization period (i.e., 1st cycle), the series resistance (R_s_) of 3D PSC was observed to be 7.66 ± 0.72 Ω. On the other hand, 2D/3D PSC was noted to have a little higher R_s_ value (11.98 ± 0.51 Ω). Since R_s_ is associated with contact resistance at ETL, HTL and the resistance originating from measurement wires and the apparatus [30], the above-mentioned R_s_ indicate that the resistance due to contact transporting layers is minor, providing high PCEs [36]. With the passage of time, an apparent increase in the R_s_ was noticed for both device types. Since external environmental conditions and measurement wires setup were kept the same throughout the experiment, R_s_ change must be related to Ω change in processes occurring at the ETL or HTL interfaces of the PSCs [32]. Regarding the charge transfer resistance R_ct_, in the case of 2D/3D PSC, its value was found to be almost half that of 3D PSC, indicating a better Au/HTM/perovskite junction. A similar observation was made from the Bode plot analysis shown in Figure 3g–i. The frequency peaks in these plots represent the time constant associated with R_ct_. As can be observed, the frequency peaks remained at higher frequencies throughout the characterization period (24 months) for 2D/3D PSC compared to the 3D samples, therefore indicating a faster charge transfer process compared to that of 3D PSC. However, the R_ct_ value of both the solar cell types increased throughout the characterization period. Nonetheless, the R_ct_ value of 2D/3D PSC stayed much lower than its 3D counterpart, indicating a stronger intermolecular connection at the HTM/perovskite interface. This is because the energy alignment of 2D perovskite is better matched with HTM than 3D perovskite. Moreover, the 2D perovskite acts as a protective layer that prevents moisture or oxygen penetration, making the HTM layer stay stable for a more extended period. In the case of 3D PSC, the HTM layer degrades with time due to moisture penetration. Therefore, it merges across the 3D perovskite, and the whole Au/HTM/perovskite interface starts acting as one block, increasing the R_ct_. The increase in R_ct_ can also be linked to the decreasing frequency peaks (f_p_) of both solar cell types (Figure 3e,f). As can be observed in Table 4 the f_p_ of 3D decreases from 198.6 Hz to 26.8 Hz, while the f_p_ of 2D/3D decreases from 252.6 Hz to 34.33 Hz. It should be noted here that the f_p_ of 2D/3D sample remains higher than that of the 3D samples throughout the characterization period. On the other hand, the recombination resistance (R_rec_) for 2D/3D PSC was much higher (almost double) than 3D PSCs at the start of the characterization, i.e., 4.06 MΩ and 2.38 MΩ, respectively. A high built-in electric field is obtained when the work is performed under short circuit conditions. Under these conditions, drift can be regarded as the dominant charge transport mode, whereas the measured resistance is considered to be dominated by charge recombination resistance [39]. A significant decrease in R_rec_ was observed with time in the case of both solar cell types, with the R_rec_ values of 3D and 2D/3D PSC being equal to 75.6 kΩ and 380 kΩ, respectively, after 24 months. However, as expected, the decrease in R_rec_ of 2D/3D PSC was much smaller than that of 3D PSC. In 3D PSC, the perovskite degrades faster, which can also be observed from the Bode plots in Figure 3e,f, where the 3D PSC starts to show a greater trend towards resistive behavior after 12 months, as indicated by a decreasing slope in the LF region in the Bode plots. A horizontal bend would indicate a more resistive behavior, representing a degraded device. As for the case of 2D/3D PSC, a negligible change was observed in the LF region slope of Bode plots, meaning that it maintained its capacitive (still with some degradation over time behavior throughout the characterization period). Degradation has a profound effect on the active layer itself, as well as on the interfacial layers of the PSCs by means of intrinsic degradation pathways as well as extrinsic factors. The intentionally designed 2D capping layer creates a relatively greater splitting in the Fermi level when deposited on top of the 3D perovskite film. Taking advantage of the deeper (dark condition) Fermi level, a larger gap is formed between the hole (Ef_p_) and electron (Ef_n_) quasi-Fermi levels under light, resulting in the optimization of energy band alignment and reduced recombination losses [40]. The recombination of electrons and holes in 2D/3D PSC might also take place at the 2D/3D interface due to charge accumulation. A built-in electric field can possibly be formed at the 2D/3D heterojunction and the electrons and holes get accumulated at this interface due to interfacial barrier of 2D/3D heterojunction, increasing recombination as a result. Therefore, it is essential to particularly focus on 2D/3D interfacial engineering in order to enhance the performance of 2D/3D PSCs. One possible technique to accomplish this is by manipulating the contacting interface of 2D/3D. Different contacting surfaces can provide different bandgap and charge recombination centers [41]. The 2D/3D heterostructures with higher bandgaps and recombination centers at 2D interface will result in higher PCEs, lower recombination rates, and higher stability. Another possible solution is by optimizing the post thermal annealing treatment process of 3D and 2D layers to obtain uniform and defect-free films.

## 3. Experimental

### 3.1. Materials and Device Fabrication

FTO was purchased from Ossila Sheffield, UK. Formamidinium iodide (FAI) and methylammonium bromide (MABr) were bought from GreatCell Solar, Queanbeyan, Australia. Lead iodide (PbI_2_) and lead bromide (PbBr_2_) were procured from TCI, Tokya, Japan, while the Cesium iodide (CsI) was purchased from abcr GmbH, Karlsruhe, Germany. To fabricate the PSCs, the FTO-coated glass substrates (3 mm thick glass substrate with an 8 Ω/sq fluorine-doped tin oxide purchased from Solaronix, Aubonne, Switzerland) were first cleaned with acetone, followed by methanol and hellmanex solution in an ultrasonic cleaner. The compact TiO_2_ layer was then deposited by spray pyrolysis at 450 °C. Titanium di-isopropoxide was diluted in isopropyl alcohol with a 1:15 volume ratio to prepare the compact TiO_2_ precursor solution. Following the compact TiO_2_ layer, a mesoporous TiO_2_ layer was spin-coated on the substrate by diluting m-TiO_2_ paste in methanol with a 1:8 weight ratio at 5000 rpm for 20 s followed by annealing at 500 °C for 30 min. Subsequently, a tin oxide passivation layer was spin-coated on the mesoporous TiO_2_ layer with 1.2% SnCl4 in water, at 3000 rpm for 30 s at 100 °C, followed by annealing at 190 °C for 60 min. Next, the triple cation 3D-perovskite precursor was prepared by diluting 0.2M MABr, 0.2M PbBr_2_, 1.15M PbI_2_, 1.1M FAI, and 1.15 M CsI in DMF and DMSO (4:1, volume ratio) mixed solvent to create [(FA_0.83_MA_0.17_)Cs_0.05_]Pb(I_0.83_Br_0.17_)_3_ perovskite layer. An amount of 37 μL of perovskite was deposited on the substrate in a glove box with around 20 to 25% humidity by a two-step spin-coating at 2000 rpm for 12 s and 5500 rpm for 30 s. An amount of 110 μL chlorobenzene was dropped on the spinning substrate after 25% completion of step 2. The sample was then annealed at 100 °C for 60 min to obtain 3D-perovskite crystals. UV ozone treatment was performed prior to each step, for 15 min. This was done to ensure any contaminants removal from the surface of the substrates. This step is strongly recommended, as it is known to enhance the interfacial connections of the different layers of PSCs and hence increases the PCE [42]. The 2D-perovskite layer (in the case of 2D/3D samples) was spin-coated on top of the 3D perovskite film to obtain a layered structure, as shown in Figure 1b. PEA+ cation was employed as the organic spacer in 2D-perovskite, since it has demonstrated impressive PCE and stability in PSCs, as discussed previously. To form an additional 2D perovskite film, 60 mM PEAI was dissolved in isopropanol and spin-coated over the 3D-perovskite film at 4200 rpm for 20 s. Next, 37 μL of the Spiro-OMeTAD precursor solution was spin-coated on the substrate. To prepare the Spiro-OMeTAD precursor solution, 1 g of spiro-OMeTAD was dissolved in 2 μL of Li-TFSI solution with acetonitrile, 2 μL of Co-TFSI solution with acetonitrile, 4 μL of TBP, and 128 μL of chlorobenzene. Lastly, the 2D/3D PSC device was completed with a thermal deposition of a 70-nm-thick gold layer. The PSCs fabricated here were prepared following the recipe described in Ref. [17]. They already performed XRD and SEM in their work in order to study and confirm the presence of the 2D layer. It is also important to mention that five samples were the reference for the 3D cells, while 20 samples were prepared in total for the 2D/3D cell.

### 3.2. Characterization of the PSCs

The PSC samples’ J-V characteristics were measured using Abet Technology SunLite Solar Simulator and by applying an external biasing using a digital source measurement unit, Keithley 2400. The J-V curves were recorded in three cycles, for the fresh as well as the aged samples over 12 months and 24 months. Next, the electrochemical impedance spectroscopy (EIS) was carried out in the dark and short-circuit conditions using Gamry Reference 3000 potentiostat, in the frequency range between 10 mHz and 1 MHz. A small AC perturbation of 10 mV was applied to allow the migration of charges. Without using any DC biasing, the actual behavior of the PSC under dark can be analyzed. Hence, the deterioration in the PSCs’ performance over time is purely the effect of the device’s degradation, without any external influences. To gain further insight from the EIS spectra, an equivalent circuit (EEC) model was developed and fitted using Gamry Echem Analyst software to quantify the different dynamic processes, charge transfer, and recombination processes in terms of resistance and capacitive elements. The experiment was repeated three times over a period of 24 months. The samples were kept in ambient room conditions.

## 4. Conclusions

In this work, I-V and EIS characterizations were carried out for 3D and 2D/3D PSCs for a period of 24 months to study the aging process of these devices. A 3RC circuit was developed to fit the obtained EIS spectra. To fit the low-frequency (LF) region of the IS spectra, the Bisquert transmission line (BTO) model was used, which accounted for the giant dielectric effect at low frequencies. The use of BTO also omitted the need to add an extra R|C for 2D/3D sample fitting and seems more realistic. It was observed from the EIS results that the HF impedance of 3D and 2D/3D PSC, which is attributed to charge transfer resistance at the Au/HTM/perovskite interface, clearly increases with aging. On the other hand, the IF response related to the recombination process at the perovskite interface undergoes a noteworthy decrease with aging for both the solar cell types. Nonetheless, the 2D/3D samples show a much slower change in its HF and IF response compared to that of 3D samples. This is an indication of the better stability of 2D/3D over 3D. From these results, it can be deduced that the 2D layer protection against environmental effects and better energy alignment of the 2D layer with HTM compared to that of 3D perovskite are the two main factors of the slower decrease in R_rec_ of 2D/3D PSC; however, it is not a complete solution in our presenting devices. In addition, the I-V characterization showed that the slope and magnitude of the current density of both the solar cell types were decreasing with aging. This was linked to reducing shunt resistance resulting in loss of current due to enhanced recombination. A minor decrease in V_oc_ was observed for both solar cell types. Recapping the above, this study revealed that the degradation process could not be entirely suppressed only by the addition of a 2D capping layer, and further work is required on the PSC layer’s engineering.

## Figures and Tables

**Table 1:** I-V characteristics and ideality factor of 3D and 2D/3D PSCs, obtained after 3 months, 12 months, and 24 months.

Sample		J_sc_, (mA/cm^2^)	V_oc_, (V)	FF	Efficiency (%)	Ideality Factor (n)
3D	1st cycle	22.96	1.09	78.17	19.44	2.03 ± 0.13
	2nd cycle	8.51	0.98	57.08	4.75	5.78 ± 0.040
	3rd cycle	6.44	0.97	43.61	2.71	6.78 ± 0.091
2D/3D	1st cycle	23.08	1.1	79.68	20.23	2.08 ± 0.22
	2nd cycle	15.32	1.01	54.14	8.10	8.28 ± 0.004
	3rd cycle	13.71	0.98	50.34	6.83	8.99 ± 0.091

**Table 2:** Parameters extracted from the log-log and semi-log plots of J-V characteristics under dark. The log-log plot was divided into four regions whose respective slopes provide critical information about the 3D and 2D/3D PSC’s trap characteristics. The semi-log plot gave information about devices’ quality using the parameter known as the ideality factor (n).

Sample	Slope (m)			
	Region I	Region II	Region III	Region IV
3D	1.22 ± 0.02	1.37 ± 0.01	5.56 ± 0.11	N/A
2D/3D	1.19 ± 0.026	1.16 ± 0.0041	3.20 ± 0.007	2.91 ± 0.021

**Table 3:** EIS parameters for 3D and 2D/3D PSCs obtained after EEC model fitting. Multiple models were evaluated; 3RC model, 3RC with Bisquert transmission line (3RC-BTO), and 3RC with an inductor and Bisquert transmission line model (Ls-3RC-BTO) and the final model (Ls-3RC-BTO) were chosen based on goodness of fit.

***3RC***										
**Sample**	**Time Period**	**R_s_**	**L_s_**	**HF Region (Charge Transfer)**			**IF/LF Region (Recombination)**			**Goodness of Fit**
		**(Ω)**	**(H)**	**R_ct_ (Ω)**	**a_ct_** **(10^−3^)**	**Y_ct_** **(10^−6^)**	**R_rec_ (Ω)**	**a_rec_** **(10^−3^)**	**Y_rec_** **(10^−6^)**	
	1st cycle	8.78 ± 0.22	-	3.03 × 10^3^	978	0.064	2.48 × 10^6^	844	2.91	7.35 × 10^−3^
3D	2nd cycle	16.0 ± 0.44	-	1.37 × 10^4^	989	0.138	1.52 × 10^5^	718	2.80	7.87 × 10^−3^
	3rd cycle	17.70 ± 0.31	-	1.51 × 10^4^	970	0.176	7.65 × 10^4^	801	3.44	4.24 × 10^−3^
	1st cycle	12.59 ± 0.26	-	1.96 × 10^3^	977	0.073	4.79 × 10^6^	910	2.55	4.33 × 10^−3^
2D/3D	2nd cycle	28.25 ± 0.45	-	1.03 × 10^4^	969	0.068	1.48 × 10^6^	859	2.15	1.65 × 10^−3^
	3rd cycle	36.91 ± 0.57	-	1.39 × 10^4^	955	0.317	3.72 × 10^5^	783	2.15	1.88 × 10^3^
***3RC-BTO***										
**Sample**	**Time Period**	**R_s_**	**L_s_**	**HF Region (Charge Transfer)**			**IF/LF Region (Recombination)**			**Goodness of Fit**
		**(Ω)**	**(H)**	**R_ct_ (Ω)**	**a_ct_** **(10^−3^)**	**Y_ct_** **(10^−6^)**	**R_rec_** **(Ω)**	**a_rec_** **(10^−3^)**	**Y_rec_** **(10^−6^)**	
	1st cycle	8.27 ± 0.26	-	3.20 × 10^3^	959	0.121	2.42 × 10^6^	853	2.83	16.7 × 10^−3^
3D	2nd cycle	16.03 ± 0.96	-	8.72 × 10^3^	963	0.130	1.02 × 10^5^	914	3.31	1.84 × 10^−3^
	3rd cycle	18.12 ± 0.43	-	1.42 × 10^4^	948	0.138	7.36 × 10^4^	749	3.25	3.59 × 10^−3^
	1st cycle	12.80 ± 0.26	-	2.07 × 10^3^	991	0.069	4.00 × 10^6^	921	2.48	3.54 × 10^−3^
2D/3D	2nd cycle	28.5 ± 0.51	-	8.27 × 10^3^	991	0.19	1.45 × 10^6^	870.5	2.16	1.31 × 10^−3^
	3rd cycle	36.92 ± 0.89	-	1.34 × 10^4^	977	0.25	3.78 × 10^5^	776	2.17	1.90 × 10^−3^
***Ls-3RC-BTO***										
**Sample**	**Period**	**R_s_**	**L_s_**	**HF Region (Charge Transfer)**			**IF/LF Region (Recombination)**			**Goodness of Fit**
		**(Ω)**	**(H)**	**R_ct_ (Ω)**	**a_ct_** **(10^−3^)**	**Y_ct_** **(10^−6^)**	**R_rec_ (Ω)**	**a_rec_** **(10^−3^)**	**Y_rec_** **(10^−6^)**	
	1st cycle	7.66 ± 0.72	1.28 × 10^−6^	3.35 × 10^3^	890	0.268	2.38 × 10^6^	821	3.9	1.08 × 10^−3^
3D	2nd cycle	16.0 ± 0.82	1.12 × 10^−6^	1.06 × 10^4^	874	0.192	1.37 × 10^5^	872	4.8	4.85 × 10^−3^
	3rd cycle	16.94 ± 0.63	1.17 × 10^−6^	1.51 × 10^4^	953	0.324	7.56 × 10^4^	783	3.61	2.57 × 10^−4^
	1st cycle	11.98 ± 0.51	7.88 × 10^−7^	2.17 × 10^3^	963	0.086	4.06 × 10^6^	922	2.49	1.36 × 10^−4^
2D/3D	2nd cycle	27.75 ± 0.62	1.38 × 10^−6^	1.05 × 10^4^	956	0.076	1.46 × 10^6^	861	2.15	2.41 × 10^−4^
	3rd cycle	36.22 ± 0.87	1.31 × 10^−6^	1.30 × 10^4^	962	0.268	3.80 × 10^5^	766	2.21	5.25 × 10^−4^

**Table 4:** Peak frequencies (f_p_) measured for 3D and 2D/3D samples with the aging process. These parameters were obtained from the Bode plots.

Sample	f_p_ (Hz)		
	1st Cycle	2nd Cycle	3rd Cycle
3D	198.6	31.3	26.8
2D/3D	252.6	79	34.3

## Supplementary Materials

The following are available online. Figure S1: log-log plot of (a, c) 3D and (b, d) 2D/3D samples. (a) and (b) are from measurements taken after 3 months. (c) and (d) are from measurements taken after 24 months.
