# Supplementary material for: Long-Term Stability Analysis of 3D and 2D/3D Hybrid Perovskite Solar Cells Using Electrochemical Impedance Spectroscopy
Source: Molecules. 2020 Dec 8;25(24):5794. doi: 10.3390/molecules25245794 (PMC7763814; doi:10.3390/molecules25245794)
Supplement: Supplementary file 1 [file molecules-25-05794-s001.pdf]

Supplementary information:

Long-term stability analysis of 3D and 2D/3D hybrid perovskite solar cells using electrochemical impedance spectroscopy

Sumayya M. Abdulrahim [1], Zubair Ahmad [1], Jolly Bhadra [2] and Noora J. Al-Thani [2]

[1] Center for Advanced Materials (CAM), Qatar University, 2713, Doha, Qatar; sumayya@qu.edu.qa and zubairtarar@qu.edu.qa

[2] Qatar University Young Scientists Center (YSC), Qatar University, 2713, Doha, Qatar; jollybhadra@qu.edu.qa and n.al-thani@qu.edu.qa@qu.edu.qa

* Correspondence: zubairtarar@qu.edu.qa (Z.A);

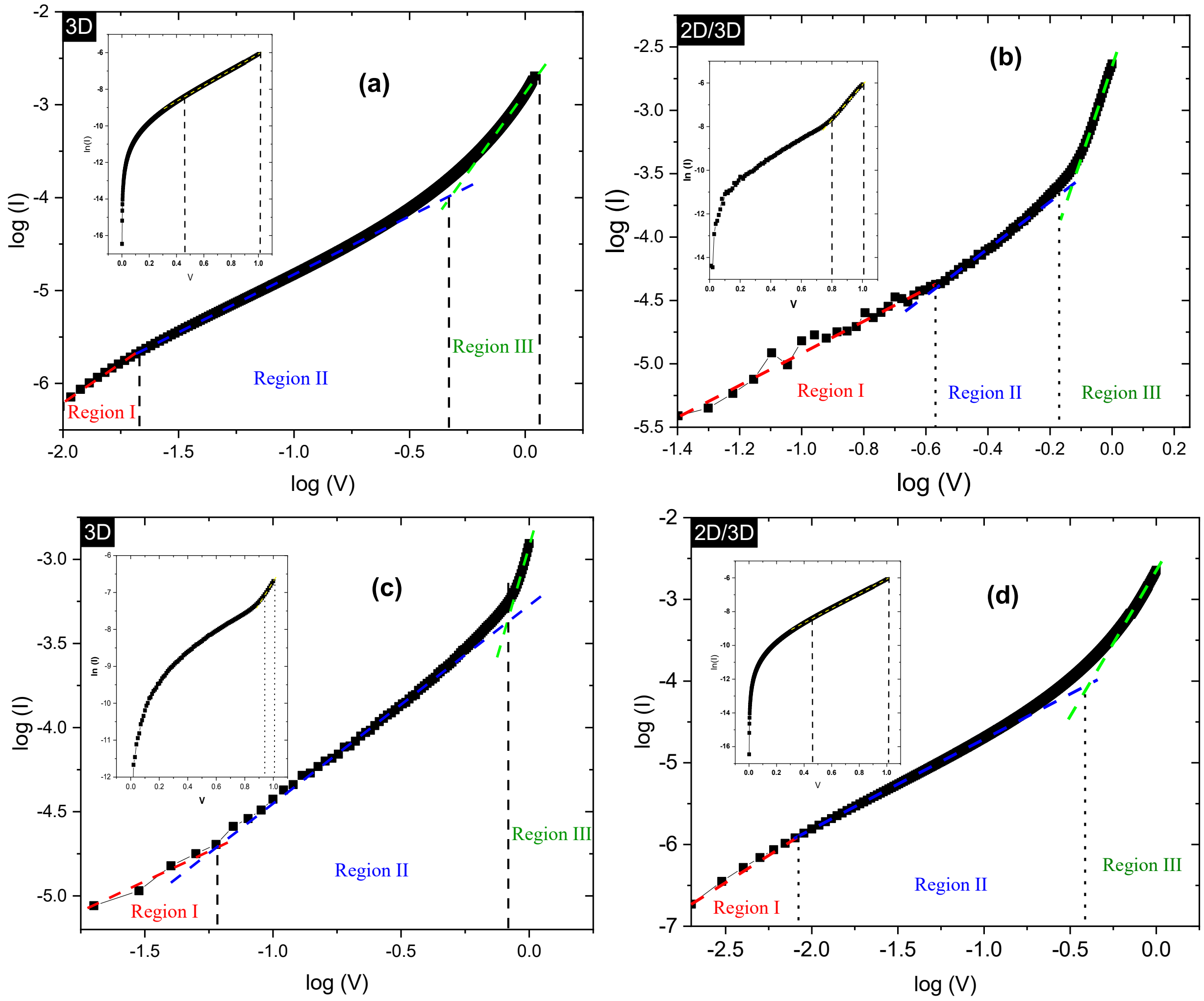

Figure S1: log-log plot of (a, c) 3D and (b, d) 2D/3D samples. (a) and (b) are from measurements taken after 3 months. (c) and (d) are from measurements taken after 24 months.
